# Variability of day-to-day pulsatility index change in children with cerebral malaria

**DOI:** 10.3389/fneur.2024.1466941

**Published:** 2024-11-11

**Authors:** Jeremy Jordan, Nicole O’Brien, Peng Li, Davin Ambitapio Musungufu, Robert Tandjeka Ekandji, Jean Pongo Mbaka, Ludovic Mayindombe, Buba Giresse, Tusekile Phiri, Sylvester June, Montfort Bernard Gushu Co, Taty Tshimanga, Karin Reuter-Rice

**Affiliations:** ^1^University of Alabama at Birmingham, School of Nursing, Birmingham, AL, United States; ^2^Children’s of Alabama, Pediatric Critical Care, Birmingham, AL, United States; ^3^Nationwide Children’s Hospital, Columbus, OH, United States; ^4^Centre Medicale Evangelique (CME), Bunia, Democratic Republic of Congo; ^5^Universite des Sciences et des Technologie de Lodja (USTL), L’Hopital General de Reference de Lodja, Lodja, Democratic Republic of Congo; ^6^Cliniques Universitaires de Kinshasa, Hopital Pediatrique de Kalembe Lembe, Universite De Kinshasa, Kimwenza, Democratic Republic of Congo; ^7^Queen Elizabeth Central Hospital, Blantyre, Malawi; ^8^Duke University, School of Nursing, Durham, NC, United States

**Keywords:** transcranial Doppler ulstrasonography, cerebral malaria, cerebral vasculature, pediatrics, pulsatility index

## Abstract

**Introduction:**

Cerebral malaria (CM) is a devastating disease and better understanding of etiologies of the resulting neurologic injury is needed. The purpose of this study is to describe the day-to-day (DTD) pulsatility index (PI) change measured by transcranial Doppler ultrasound (TCD), a novel measure of cerebral and vascular changes, in children with CM.

**Methods:**

A retrospective analysis of 122 children in sub-Saharan Africa with CM and 3 or more sequential TCD measurements was performed. Variability of DTD PI change was calculated as a measure of changes in vasculature overtime. Neurologic outcome was determined by the Pediatric Cerebral Performance Category (PCPC) score, a measure of neurologic function.

**Results:**

Of the 122 participants, 77.9% had a good neurologic outcome (no neurologic sequelae), and 22.1% had a poor outcome (neurologic sequelae or died). Patients who had a poor neurologic outcome had higher levels of variability of DTD PI change in the right middle cerebral artery (MCA) (0.14 ± 0.21) and left MCA (0.17 ± 0.41) compared to those who had a good neurologic outcome (0.1 ± 0.1 and 0.11 ± 0.19, respectively). A higher variability of both left and right MCA DTD PI change was also associated with higher brain volume assessed through neuroimaging.

**Discussion:**

Variability of DTD PI change may provide early prognostic information regarding PCPC outcomes and brain volume changes seen in CM patients. Expanded research on pathophysiologic contributors to variability of DTD PI changes in children with CM is warranted.

## 1 Introduction

In 2022, there were > 600,000 reported deaths from malaria worldwide (1). Over 95% of malaria deaths globally occurred in Africa in children under 5 years (1). Cerebral malaria (CM) is a severe form of the disease where coma not attributable to another cause occurs in an individual with Plasmodium falciparum parasitemia (2, 3). Infected erythrocytes attach to the endothelial cells of the microvasculature, something known as parasite sequestration (4–7). The use of artesunate and preventative techniques have decreased deaths from malaria, however, additional adjunctive treatments have failed to further reduce mortality and morbidity (8, 9). Up to half of individuals who survive CM have neurologic sequelae such as epilepsy, behavioral dysregulation, motor delays, and cognitive impairments (10, 11). Further exploration is needed to determine how cerebral malaria results in profound neurologic injury given the parasite remains in the vasculature and does not enter the brain parenchyma. Once determined, the impact of the deployment of targeted treatments may be undertaken (12).

Transcranial Doppler (TCD) ultrasound is a portable, safe, and non-invasive imaging technique that is readily available in many African hospitals which measures cerebral blood flow velocities (CBFV) in all major vessels of the cerebral arterial circulation (13, 14). Emerging research in CM utilizing TCD has demonstrated five distinct phenotypes of abnormal CBFV and waveform patterns. These phenotypes include hyperemia, low flow, microvascular obstruction, vasospasm, and isolated posterior circulation high flow (15). These phenotypes demonstrate that TCD can be used to characterize changes in the neurovasculature children with CM. Additionally, O’Brien et al. noted that these changes in TCD findings are likely due to primary pathologic changes and not other typical physiologic alterations (15). For example, fever, seizures, and hypercapnia were not associated with hyperemia whereas non-invasive markers of increased intracranial pressure (ICP) were not associated with low flow. Further work is needed to determine aspects of TCD measurements or waveforms that provide additional understanding of pathophysiologic underpinnings of these phenotypes.

The Gosling pulsatility index (PI) is a TCD parameter calculated as the difference between systolic CBFV and diastolic CBFV divided by the mean CBFV [CBFV_sys_ – CBFV_dia_/CBFVmean] (16). An increased PI could be the result of increases in systolic flow velocity and/or decreases in diastolic flow velocity. Inversely, a decrease in PI is seen when systolic flow velocity decreases and/or diastolic flow velocity increases. The former is commonly seen as a result of increased ICP where diastolic flow, due to decreased driving pressure during this phase of the cardiac cycle, is preferentially decreased. Asil et al. found that, in patients with MCA infarction, the PI of the MCA increased over the first three days and was correlated with the significance of midline shift on the third day after stroke (17). Studies have demonstrated that a PI ranging from 1.3 to 1.5 correlates with some degree of intracranial hypertension (18). In patients with traumatic brain injury, PI values significantly decrease after decompressive craniectomy (19). Alternatively, the PI is reduced in vascular changes such as vasospasm where autoregulation results in decreased resistance to flow, and an increased diastolic flow velocity. As such, PI has clinical applications in the assessment of cerebral circulation (20).

While a single PI value provides a measure of underlying cerebrovascular physiology during the examination, it does not capture dynamic changes over time that could provide more insight into underlying pathologic changes. A novel use of PI has been developed, referred to as Day-to-Day (DTD) PI change, which evaluates the variability in PI from one day to the subsequent day (21). As a measurement of spread between the mean, variability demonstrates the level of stability of PI rather than simply a high or low average value. In a study of 42 children with traumatic brain injury, Jordan et al. found that variability of DTD PI change was larger in children with worse neurocognitive outcomes, irrespective of injury severity (21). Presumably the high variability of DTD PI change indicates a loss or defect in autoregulatory functioning of the cerebral vasculature which leads to worse neurocognitive outcomes due to periods of inappropriate perfusion.

Therefore, we hypothesized that the variability of DTD PI change is a potential measure that will further understanding of the alterations to the cerebral vasculature in pediatric cerebral malaria patients. We therefore performed a retrospective analysis of 122 children in sub-Saharan-Africa with CM and described the association between variability of DTD PI change and brain volume score, neurologic outcome, and TCD derived CM phenotypes.

## 2 Materials and methods

After Institutional Review Board approval and execution of a Data-Use Agreement, a de-identified data set from parent study was transferred to a secure database. The parent study enrolled children with cerebral malaria who were treated at Queen Elizabeth Central Hospital in Blantyre, Malawi, Kalembe Lembe Children’s Hospital in Kinshasa, Democratic Republic of the Congo (DRC) and Lodja District Referral Hospital in Lodja, DRC between January 2018 and June 2021. Inclusion criteria included: age 6 months – 12 years, met the World Health Organization definition of cerebral malaria (Plasmodium falciparum parasitemia, Blantyre Coma Score (BCS) ≤2, and no other discernible cause of encephalopathy). Exclusion criteria included developmental delay, diagnosis of sickle cell disease, severe malnutrition, and advanced HIV disease. Children with developmental delay were excluded because the outcome measurement is validated on children without developmental delay. Children with sickle cell disease have a high frequency of abnormal TCD examinations as a population. Additionally, the impact of severe malnutrition and/or advanced HIV status on TCD examinations is unknown. Therefore, these children were excluded. This study utilizes the same inclusion and exclusion criteria with an additional exclusion criterion. To calculate variability of DTD PI change, at least three sequential PI measurements are required; therefore, participants in the parent study without three sequential PI measurements were excluded.

Data was imported into Excel for cleaning. R software (R Core Team, 2022) was used for the data analysis. Descriptive statistics were used to explore patient and disease characteristics. The participant’s DTD PI change and subsequent analysis was calculated using the following steps.

*Step 1* – DTD PI change is defined as the difference in PI measurement from one day to the next and is calculated using the following formula (using day1 and day2 as an example):

(PIday2 – PIday1) / PIday1 = DTD PI change.

The DTD PI change for other continuous days was calculated similarly.

*Step 2* – These values are averaged to derive their Mean DTD PI change during the hospital stay. This value represents the mean difference between each consecutive day’s measurement (21, 22).

*Step 3* –The variability of DTD PI change, defined as the dispersion of PI measurements from their mean values was then calculated using the variation function. As a measurement of spread between the mean, variation demonstrates the level of stability of PI rather than simply a high or low average value. Values were calculated for both MCAs in each participant.

*Step 4* – Simple linear regression is used to explore the association of neurocognitive outcomes with variation of DTD PI change, and the association with Brain Volume Score (BVS).

*Step 5* – Lastly, the variability of DTD PI change is explored by TCD phenotypes.

Brain Volume Score (BVS) is a classification of brain size based on neuroimaging. BVS ranges from 1, marked atrophy, to 8, effacement of the cisterns and sulci with herniation. A score of 3 indicates normal brain volume (23). Neurocognitive outcomes were classified as normal, sequelae, or dead as determined by the Pediatric Cerebral Performance Categorization (PCPC) score at hospital discharge. The PCPC scoring system was developed to measure morbidity after critical illness in the pediatric population (24, 25) The scores progress from 1 to 6 to represent increasing levels of impairment. Children with a PCPC score of 1 were classified as having a ‘normal’ outcome, a score of 2–5 was classified as ‘sequelae’ due to the neurologic impairment, and a score of 6 was classified as ‘dead’.

## 3 Results

The participants with 3 or more sequential TCD (*n* = 122) were predominantly male (51.6%) with an average age of approximately 5 years old (5 ± 2.5 years). Additionally, 68 (55.7%) participants received a magnetic resonance imaging scan. Of these 68 participants, a majority had a BVS of 5 or 6 (27 (40%) and 17 (35%), respectively). Of the 122 participants, 95 (77.9%) had a good neurologic outcome (no neurologic sequelae), and 27 (22.1%) had a poor outcome (neurologic sequelae or died). See Table 1 for full demographic information.

**Table 1:** Demographic information.

Demographic Information	*n* = 122
Age (months), mean (SD)	60.4 ± 30.1
Male, *n* (%)	63 (51.6%)
Blantyre Coma Score on Admission, *n* (%)	
0	17 (13.9%)
1	49 (40.2%)
2	56 (45.9%)
3	0 (0%)
4	0 (0%)
5	0 (0%)
Brain Volume Score, *n* (%)	*n* = 68
2 – Atrophy	1 (1%)
3 – Normal	4 (6%)
4 - Slightly increased volume	6 (9%)
5 – Mildly increased volume	27 (40%)
6 – Moderately increased volume	17 (35%)
7 – Substantially increased volume	10 (15%)
8 – Effacement of sulci and cisterns with herniation	3 (4%)
Outcome, *n* (%)	*n* = 122
Good	95 (77.9%)
Sequelae	27 (22.1%)
CM phenotype, *n* (%)	*n* = 122
Hyperemia	40 (32.8%)
IPH	18 (14.8%)
Low flow	29 (23.8%)
Micro-obstruction	8 (6.6%)
Vasospasm	7 (5.7%)
Normal	5 (4.1%)
Mixed	15 (12.3%)

Analysis of the outcome groups revealed that patients who had poor neurologic outcome had higher variability of DTD PI change in the right MCA (0.14 ± 0.21) compared to those with a good neurologic outcome (0.1 ± 0.1), with a difference of 0.03 (95% CI [−0.13, 0.05]). In the left MCA, the variability of the DTD PI change was also higher in those with poor outcomes (0.17 ± 0.41) compared to those with good neurologic outcomes (0.11 ± 0.19), with a difference of 0.06 (95% CI [−0.23, 0.11]). See Table 2 for more information.

**Table 2:** Day-to-Day PI Change by Neurologic Outcome.

DTD PI change by outcome	Good outcome	Poor outcome	Difference of the means (95% CI)
Variability of DTD PI Change LMCA, mean (SD)	0.11 (SD = 0.19)	0.17 (SD = 0.43)	0.06 (−0.23, 0.11)
Variability of DTD PI Change RMCA, mean (SD)	0.1 (SD = 0.11)	0.14 (SD = 0.21)	0.03 (−0.13, 0.05)

When evaluating variability of DTD PI change by BVS, participants with a higher BVS score had a higher variability of DTD PI change. This difference in variability of DTD PI change was seen in both left and right MCAs. In the left MCAs, the most frequent BVS of 5 equated to a lower DTD PI change compared to a BVS of 8 (0.053 ± 0.077 and 0.898 ± 1.197, *p* = 0.012, respectively). In the right MCAs, this pattern again appeared in participants with a BVS of 5 compared to BVS of 8 (0.075 ± 0.1 and 0.502 ± 0.516, *p* = 0.189 respectively). See Table 3 for more information.

**Table 3:** Day-to-day PI change by BVS.

DTD PI change by BVS	Brain volume score							ANOVA *p*-value
	2 (*n* = 1)	3 (*n* = 4)	4 (*n* = 6)	5 (*n* = 27)	6 (*n* = 17)	7 (*n* = 10)	8 (*n* = 3)	
Variability of DTD PI change LMCA, mean (SD)	0.041 ± 0	0.18 ± 0.185	0.042 ± 0.042	0.053 ± 0.077	0.127 ± 0.172	0.151 ± 0.24	0.898 ± 1.197	*p* = 0.012
Variability of DTD PI change RMCA**, mean (SD)	0.028 ± 0	0.218 ± 0.165	0.188 ± 0.2	0.075 ± 0.1	0.143 ± 0.195	0.121 ± 0.066	0.502 ± 0.516	*p* = 0.189

DTD, Day-to-day; PI, Pulsatility index; LMCA, left middle cerebral artery; RMCA, right middle cerebral artery; BVS, Brain volume score.

Lastly, the variability of the DTD PI change demonstrated discreet differences in the previously described CM phenotypes. The largest variability of DTD PI change was seen in the microvascular-obstruction group for both the LMCA (0.478 ± 0.833, *p* = 0.019) and RMCA (0.237 ± 0.386, *p* = 0.273). The smallest variability of DTD PI change was seen in the group in the normal phenotype group for both the LMCA (0.048 ± 0.038, *p* = 0.019) and RMCA (0.054 ± 0.064, *p* = 0.273). See Table 4 for information on all phenotypes.

**Table 4:** Day-to-day PI change by phenotype.

DTD PI change by phenotype	Hyperemia	IPH	Low flow	Micro obstruction	Normal	Vasospasm	ANOVA *p*-value
Variability of DTD PI change LMCA, mean (SD)	0.119 ± 0.247	0.093 ± 0.119	0.106 ± 0.125	0.478 ± 0.833	0.048 ± 0.038	0.073 ± 0.088	*p* = 0.019
Variability of DTD PI change RMCA, mean (SD)	0.097 ± 0.11	0.093 ± 0.118	0.117 ± 0.123	0.237 ± 0.386	0.054 ± 0.064	0.163 ± 0.27	*p* = 0.273

DTD, Day-to-day; IPH, Isolated posterior circulation high flow; PI, Pulsatility index; LMCA, left middle cerebral artery; RMCA, right middle cerebral artery.

## 4 Discussion

In a group of 122 pediatric participants with CM who had daily TCD measurements of their bilateral MCAs, the variability of DTD PI change was evaluated in the context of neurologic outcome, BVS, and TCD phenotype. Individuals with the normal phenotype have the lowest variability of DTD PI change indicating low neurovascular involvement. These participants also had better outcomes indicating perhaps that they had less significant disease at the time of presentation or may have had an alternate diagnosis. Participants with hyperemia, isolated posterior hyperemia, vasospasm or low flow may be experiencing abnormal vascular tone. This dysregulated vascular tone may account for the lower variability of DTD PI change compared to a more volatile phenotype such as micro-obstruction. In the micro-obstructive phenotype, the primary pathophysiologic mechanism is hypothesized to be sequestered red cells occluding the microcirculation. Artesunate results in rapid parasite clearance, which may cause a highly abnormal PI to quickly return to normal as parasitized RBCs are cleared and result in a high DTD PI change.

Participants with a higher BVS had higher variability of DTD PI change. While brain volume is likely influenced by the factors noted above, identifying alternate measures that are more easily obtainable than MRI in resource limited settings where malaria is endemic is important as BVS is an important prognostic marker. Therefore, the use of TCD may be able to provide similar information portably, non-invasively, and in real time. Future studies with larger sample sizes should assess this relationship.

TCD has been used to evaluate the cerebrovasculature in several cohorts of children with CM (12, 15). Multiple different TCD abnormalities were described in children with CM with studies classified into one of five distinct phenotypes. In the current study, each of the phenotypes had a distinct variability of DTD PI change (Table 4). In this participant group, 22.1% of the participants died or had neurologic sequelae after treatment for CM. These participants demonstrated a higher variability of DTD PI change. DTD PI variability can be increased in the setting of brain parenchymal changes (such as cerebral edema, cerebral hemorrhage) that increase ICP, in alterations to vascular tone, or in venous congestion. In CM, a significant systemic inflammatory response occurs, cytokines are released that damage the blood–brain barrier, and a secondary vasogenic edema has been reported to occur (26). Parasite sequestration may result in cerebral blood flow disruption with cytotoxic edema. Lastly, venous congestion occurs as infected red blood cells (RBC) obstruct microvasculature and impair forward flow and venous drainage. The poor outcome of these individuals with high variation of DTD PI change may be due to these factors that in isolation or together lead to intracranial hypertension.

This study describes the first application of DTD PI change in this population and begins to lay the foundation for clinical changes. As a portable and low-cost imaging modality that is available in many low and middle resourced countries, TCD is a valuable tool in clinical care (13, 14). This study adds to the early research on DTD PI change and its use in pediatric neurologic disorders as well as demonstrates its feasibility (21). While additional research is needed to develop formal clinical guidelines, this study demonstrates the importance of continued research in this area to determine feasibility of more frequent TCD measurements, the optimal timing of the measurements, and ideal interval between measurements.

### 4.1 Limitations

This relatively small sample size of 129 patients and retrospective design limits our ability to generalize these findings. Children with less than 3 days of sequential TCD measurements were necessarily excluded further limiting the sample size. Thus, variability of DTD PI change was not available in those who recovered quickly or died soon after admission, thereby, further limiting the sample. TCD was only performed while Blantyre Coma Score was <3. Additionally, the data is a convenience sample of patients who presented with C.M. who had TCDs performed as a component of their care. Lastly, as some findings were not statistically significant, additional research is needed before clinical recommendations can be made. Future research should focus on larger sample size, more frequent TCD studies, account of age, sex, and time of day, and continued TCD studies on all participants including those that recover or die quickly.

## 5 Conclusion

DTD PI change may help to prognosticate the neurologic outcomes in children with CM. The pathophysiologic underpinnings of increased DTD PI variability in those with poor outcomes should be explored as TCD could then be used as a point of care neuromonitoring tool and inform treatment strategies to improve outcomes.

## Data Availability

The original contributions presented in the study are included in the article/supplementary material, further inquiries can be directed to the corresponding author.
